# Dendritic Cells Promoted by Ginseng Saponins Drive a Potent Th1 Polarization

**DOI:** 10.4137/bmi.s585

**Published:** 2008-04-18

**Authors:** Masao Takei, Eiichi Tachikawa, Akemi Umeyama

**Affiliations:** 1 Division of Cellular Allergology, Research Center Borstel, Parkallee 22, D-23845, Germany; 2 Department of Pharmacology, Iwate medical University, Uchimaru 19-1, Morioka 020-8505, Japan; 3 Faculty of Pharmaceutical Sciences, Tokushima Bunri University, Yamashiro-cho, Tokushima 770–8514, Japan

**Keywords:** dendritic cells, terpenoids, ginseng saponins, Th1 response, CTL

## Abstract

Dendritic cells (DC) play a pivotal role in the initiation of T-cell-mediated immune responses, making them an attractive cellular adjuvant for use in cancer vaccines. The interaction of T cells with DC is crucial for directing T cell differentiation towards the Th1, Th2 or Th17 type, and several factors determining the direction of the T cell polarization. IL-12 plays a central role in the immune system, not only by augmenting the cytotoxic activity of T cells and NK cells and regulating IFN-γ production, but also by the capacity of IL-12 to promote the development of Th1 cells. Therefore, it is important to identify factors that might affect the differentiation, maturation and function of DC. Ginseng is a medicinal herb widely used in Asian countries, and many of its pharmacological actions are attributed to the ginsenosides. Moreover, T-cadinol and calamenene are sesquterpenes isolated from the heartwood of *Cryptomeria japonica* being pharmacologically active substances. We investigated whether M1 and M4, end products of steroidal ginseng saponins metabolized in digestive tracts, as well as T-cadinol and calamenene can drive DC maturation from human monocytes *in vitro*. Human monocytes were cultured with GM-CSF and IL-4 for 6 days under standard conditions, followed by another 2 days in the presence of M1, M4, T-cadinol or calamenene. The expression levels of CD1a, CD80, CD83, CD86 and HLA-DR on M1-primed DC, M4-primed DC, T-cadinol-primed DC and calamenene-primed DC were enhanced with a concomitant decrease in endocytic activity. M1-primed DC, M4-primed DC, T-cadinol-primed DC or calamenene-primed DC enhanced the T cell stimulatory capacity in an allo MLR (allogeneic mixed lymphocyte reaction). Naïve T cells co-cultured with allogeneic M1-primed DC, M4-primed DC, T-cadinol-primed DC or calamenene-primed DC turned into typical Th1 cells, which produced large quantities of IFN-γ and released small amounts of IL-4 depending on IL-12 secretion. In the CTL assay (cytotoxic T-lymphocyte assay), the production of IFN-γ and ^51^Cr release on M4-primed DC was more augmented than of immature DC or TNF-α-primed DC. These results suggest that M1, M4, T-cadinol and calamenene appear to be a good factor to induce DC maturation, or even better in some respect, for the use in clinical DC therapy to induce strong Th1 type immune responses.

## Introduction

Dendritic cells (DC) are considered to be the principal antigen presenting cells (APC), whose main function is to identify microbial structures and present these to naïve T cells in the lymph node [1–3] and clinical application of DC has been initiated as a cellular immunotherapy against cancer [4]. DC are generated from either myeloid or lymphoid bone marrow progenitors through intermediate DC precursors that home to sites of potential antigen entry, where they differentiate locally into immature DC. After antigen capture in the presence of maturation signals associated with inflammation or infection, immature DC are activated by TLRs, interferon’s (IFNs), or members of TNF-R family and undergo a complex maturation process. This process *in vivo* is paralleled by migration of DC to T cell-rich areas of lymphoid organs, where they present antigen-derived peptides to antigen-specific T-cells and direct their differentiation into T effector or memory cells [5]. The interaction of T cells with DC is crucial for directing T cell differentiation towards the Th1, Th2 or Th17 type and several factors determine the direction of T cell polarization [6–12]. Human-monocytes-derived DC matured by either interferon-alpha (IFN-α), tumor necrosis factor-alpha (TNF-α) or LPS in vitro produce high levels of IL-12 and induce Th1 cells [13–15]. Th1 responses predominate in organ-specific autoimmune disorders, acute allograft rejection and in some chronic inflammatory disorders [16] and Th2 responses predominate on Omenn’s syndrome, transplantation tolerance, chronic graft-versus-host disease and allergic diseases [17]. Th17 cells comprise a recently identified Th-cell lineage that has a pro-inflammatory role in autoimmunity and tissue inflammatory [18]. One of the most important goal of DC research is the development of DC-based strategies for enhancing immune responses against tumors and infectious agents. A variety of preparations of DC can stimulate anti-tumor immunity, including DC loaded with proteins, DC fused with tumor cells and DC transduced with tumor-derived DNA or viral vectors. Analyses of the clinical results suggest that the maturation status of DC used in such protocols greatly affects the immune response that follows the treatments. Thus, for immunotherapeutic applications, it appears crucial to identify factors that might affect the differentiation, maturation, and function of DC. Therefore, it is important to identify factors that might affect the differentiation, maturation and function of DC.

**Figure 1:**
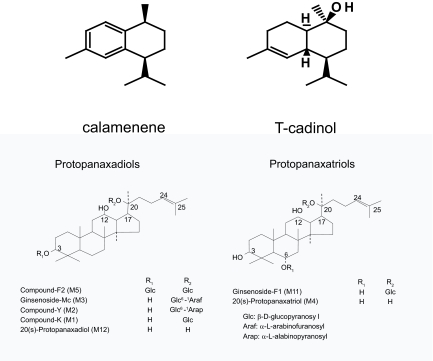
Structures of M1, M4, T-cadinol and calamenene.

The root of Panax ginseng C.A. Meyer has been widely and well used as an important of many Chinese prescriptions called “Kan-Pou medicine” for more than 2000 years and is now well known as CAM (complementary and alternative medicine) throughout the world. Recently, some reports have shown that the ginseng and its major component (saponins) ameliorate the symptoms and the lesions evoked by stress (anti-stress action), for example, improvements in gastrointestinal symptoms (anorexia, dyspepsia, etc.), gastric ulcer, fatigue, boredom, anxiety and essential hypertension in stressed animals and human [19–21]. Moreover, Yun [22] has shown that the prolonged administration of ginseng extract significantly inhibited the incidence of hepatoma and also proliferation of pulmonary tumors induced by aflatoxin B1 and urethane. It has been learned that the ginsenosides in ginseng are metabolized by gastric acid and by enzymes in intestinal bacteria when they are orally administered [23, 24]. The sugar moieties of saponins are in turn hydrolyzed in the digestive tracts. The ginsenosides are classified in three groups, the protopanaxadiol, the protopanaxatriol and the oleanoic acid groups, on the basis of the chemical structures of their aglycones. Most of the ginsenosides belong to the protopanaxa-type (steroidal saponins), and the major metabolites of ginseng including the intermediates are M1, M2, M3, M5 and M12 derived from the protopanaxadiols and are M4 and M11 from the protopanaxatriols. We have recently reported that M4 inhibited the ACh-induced secretion of catecholamines from bovine adrenal chromaffin cells and this inhibition was due to the blockade of Na^+^influx through nicotinic ACh receptor-operated cation channels [25]. Thus, ginseng saponin metabolites have already been proven to be bioactive substances. Because of its wide immunomodulatory properties, DC might be a potential target for the ginseng and its major components. On the other hand, T-cadinol and calamenene, which are phytochemically classified as sesquiterpene, are isolated from the black heartwood of *Cryptomeria japonica*. T-cadinol is isolated from scented myrrh, which is the resin of the plant *Commiphora guidottii* Chiov., Burseraceae [26]. This resin is widely used in Somalia traditional medicine as remedy for treating various gastrointestinal disorders and diarrhea [27]. T-cadinol has been shown to inhibit CT (Cholera toxin)-induced intestinal hypersecretion in mice and electrically induced contractions of the isolated guinea pig ileum. Zygmunt et al. [28] have shown that T-cadinol is a calcium antagonist at high doses and interacts with dihydropyridine binding sites on the voltage-operated calcium channels. Thus, T-cadinol and related compounds have also pharmacologically activity, but its immunomodulatory properties are still unknown. In this study, we investigated the ability of M1 and M4 (Fig. 1), endproducts of steroidal ginseng saponins metabolized in digestive tracts, as well as T-cadinol and calamenene (Fig. 1) to influence the differentiation of DC from peripheral blood monocyte.

## Materials and Methods

### Culture medium, reagents and monoclonal antibodies

The culture medium used in this study was serum-free AIM-V medium (Life Technologies, Paisley, U.K.). Recombinant human IL-4 (IL-4), recombinant human TNF-alpha (TNF-α), recombinant human granulocyte-macrophage colony-stimulation factor (GM-CSF) and CD40-L were purchased from R and D Systems (Minneapolis, MN). LPS from *Escherichia Coli* was purchased from Sigma-Aldrich (St. Louis, MO). CT was purchased from Boehringer Mannheim GmbH (Germany). For flow cytometry, monoclonal antibodies (mAbs) towards the following antigens were purchased from Becton Dickinson (San Jose, CA): anti-CD14-FITC, anti-CD1a-PE, anti-CD80-PE, anti-CD83-PE, anti-CD86-PE and HLA-DR-FITC. Endotoxin levels in all agents were below 1.0 EU/ml.

### Ginseng saponins, and isolation of T-cadinol or calamenene from the Black Heartwood of *C. japonica*

The ginseng saponins were supplied by the Korea Tobacco and Ginseng Corp. (Seoul, Republic of Korea). The purities of the ginsenosides were checked by the thin-layer chromatography and nuclear magnetic response according to the method of Kawashima and Samukawa [29] and the purity of samples was >95%. The metabolites of the ginseng saponins (M1 and M4)) were prepared as previously described [25]. T-cadinol and calame-nene were prepared as previously described [30]. The purity of T-cadinol and calamenene was >99%. The metabolites of the ginseng saponins (M1 and M4), T-cadinol and calamenene were dissolved in dimethylsulfoxide. The concentration of dimethylsulfoxide in the culture medium was 0.1%, which had no effect upon the culture and the production of cytokines under the conditions used in this study. The endotoxin in M1, M4, T-cadinol and calamenene was removed using Endo Trap 5/1 (Endotoxin removal system, Profos AG, Regensburg, Germany). Leading to LPS concentrations below the detection limit of the assay (<0.05 EU/ml). LPS-depleted M1, M4, T-cadinol and calamenene were used for subsequent experiments.

### Generation of monocytes-derived DC

All cell subsets were isolated from peripheral blood of normal healthy donors. PBMC were first isolated from heparinized whole blood by Ficoll/Isopaque/1.077 g/ml (Pharmacia, Freiburg, Germany) density gradient centrifugation (465 × g, 45 min, 22 °C) as previously described [31]. PBMC were further separated into monocytes and lymphocytes by counterflow centrifugation using the JE-6B-elutriator system (Beckman Instruments Inc., Palo Alto, CA) [31]. The purity of CD14+ monocytes was always more than 90%. Monocytes were cultured with GM-CSF (25 ng/ml) and IL-4 (25 ng/ml) in serum-free AIM-V medium for 6 days. At day 6, cells (>95% CD1a+, CD14−) were harvested and re-cultured in serum-free AIM-V medium containing GM-CSF and IL-4 for additional 2 days with various concentrations of M1, M4, T-cadinol or calamenene, but with LPS (100 ng/ml), CT (10 μg/ml) or TNF-α (25 ng/ml). All subsequent tests were performed after harvesting the cells at day 8 and after removing the above factors by extensive washing. The medium was replenished with cytokines every 2 days. To determine the production of IL-6, IL-10 and IL-12p70 by M1-, M4-, T-cadinol-, calamenene-, LPS-, CT- or TNF-α-primed DC, DC (4 × 10^4^ cell/well) were stimulated with CD40-L (3.0 μg/ml) for 24 h. The cell-free supernatants were collected and frozen at −20 °C until measurement of cytokines using enzyme-linked immunosorbent assay (ELISA).

### Immunophenotype studies

Dual-Colour immunofluorescence was performed using the following panel of monoclonal antibodies: PE-conjugated antihuman CD1a, FITC-conjugated antihuman CD14, PE-antihuman CD80, PE-antihuman CD83, PE-antihuman CD86 and FITC-HLA-DR. Negative control was isotype-matched with irrelevant monoclonal antibodies (Becton Dickinson). Cells were re-suspended in staining medium containing PBS, 5% BSA and 0.1% NaN_3_, and then fixed with 1% paraformal-dehyde. Isotype controls were run in parallel. Cell debris was eliminated from the analysis by forward and side scatter gating. The samples were analyzed on FACSCalibur (Becton Dickinson) with Cell-Quest software (Becton Dickinson). Ten thousand cells were analyzed per sample. The results were expressed as MFI.

### Allo MLR

CD4+ naïve T cells for the allo MLR were obtained from allogeneic PBMC using magnetic cell sorting and separation of biomolecules (MACS) beads (Miltenyi Biotec). The purity of isolated cells was >95% of CD4+ naïve T cells as determined by flow cytometry using FACSCalibur. Allogeneic CD4+ naïve T cells (5 × 10^4^ cell/well) were co-cultured in 96-well round-bottomed culture plates with graded doses (2 × 10^2^ to 5 × 10^4^) of irradiated (30 Gy) DC. After 5 days, cells were pulsed with 1μCi [^3^H]-methylthymidine per well for 16 h, then harvested and analyzed in a liquid scintillation counter.

### Determination of naïve T cell polarization by DC

Irradiated (30 Gy) DC were co-cultured with naïve T cells (2.5 × 10^5^ cell/200 μl) at 1:5 DC/T cell ratio in 96 well U-bottomed tissue culture plates (Costar, Cambridge, MA). Some cultures were supplemented with neutralizing Abs to block endogenous cytokines: anti-IL-12 (10 μg/ml, R and D Systems). On day 5, cells were washed extensively and expanded with fresh medium containing 10 U/ml of recombinant human IL-2 (IL-2) (Shionogi Pharmaceutical Company, Osaka, Japan). One hundred microliters of culture supernatant was replaced with medium of the same concentration every 3 days. On day 14, cells were washed, counted, and T cells (10^6^/ml) were re-stimulated for 24 h on plates coated with anti-CD3 (0.2 μg/ml; BD-Pharmingen) and anti-CD28 (2.0 μg/ml; BD-Pharmingen). The cell-free supernatants were collected and frozen at −20 °C until measurement of cytokines using ELISA.

### Intracellular cytokine staining

The intracellular cytokine concentrations of the harvested T cells were measured by FACS analysis as previously described [32]. Briefly, T cells (10^6^/ml) were stimulated with phorbol myristate acetate (PMA) (10 ng/ml) and ionomycin (1 μg/ml) for 5 h at 37 °C in a water bath. Brefeldin A (10 μg/ml) was added during the last 2 h of incubation to prevent cytokine secretion. Cells were collected, fixed with 1% paraformaldehyde, permeabilized with a commercial solution (BD-Pharmingen), and stained with FITC-labelled anti-IFN-γ (IgG_2a_) and PE-labelled anti-IL-4 (IgG_1_) mAbs. The samples were analyzed on a FACS-Calibur with CellQuest software. Ten thousand cells were analyzed per sample.

### Transmigration assay

Migration of DC induced by macrophage inflammatory protein (MIP)-3β was measured using a double chamber system as previously described [33]. Briefly, Transwells of 5 μm pore size filters (Kurabo, Osaka, Japan) were used. MIP-3β was seeded at concentrations of 0.1, 1.0, 10 and 100 nM diluted with 500 μl of culture medium in the lower chambers of 24-well plates. DC (1 × 10^5^/100 μl) were then added to the upper chambers of the Transwell plates. Transwell cultures were maintained for 5 h in 5% CO_2_ at 37 °C, and DC migrated from the upper wells were counted using a Colter Counter. The results were expressed as net migration percent calculated as:(the number of cells that migrated into the lower chamber containing chemokine-number of cells that migrated in medium alone)/total number of cells loaded in the upper chamber × 100.

### Measurement of intracellular Ca^2+^ concentration

The determination of intracellular Ca^2+^ ([Ca^2+^]i) concentration was carried out as previously described [34]. Briefly, 2 × 10^6^ cells were incubated for 45 min at 37 °C with 2.5 μM of fura-2/AM in 5 mM HEPES buffer containing 140 mM NaCl, 4 mM KCl, 1.25 mM CaCl_2_, 1 mM Na_2_HPO_4_, 1 mM MgCl_2_, 11 mM gulcose and 0.1% bovine serum albumin at pH 7.4. Samples of the cell suspension were placed in the cuvette. Then, MIP-3β (100 nM) was added with a microsyringe directly into the cuvette, without interrupting the recording. [Ca^2+^]i was monitored with a CAF110 spectrofluorimeter (λex = 340 nm and 380 nm, λem = 510 nm; JASCO, Tokyo).

### Reverse transcription-polymerase chain reaction (RT-PCR) for CCR7

Total RNA was extracted from 1–2 × 10^6^ DC using an RNeasy Mini Kit (Qiagen, Germany). The cDNA synthesized from total RNA by Moloney murin leukaemia virus reverse transcriptase (Stratagene, Austin, TX, U.S.A.) was subjected to RT-PCR using 30 cycles at 94 °C for 0.5 min, 57.5 °C for 1 min, and 72 °C for 1 min for CCR7 (Takara, Tokyo, Japan). The sense and anti-sense oligonucleotide primers for CCR7 were 5′-CGCGTCCTTCTCATCAGCAA-3′ and 5′-GTCCCGACAGGAAGACCACT-3′, respectively. PCR products were identified by electrophoresis on 2% agarose gels that were photographed.

### Cytotoxic T-lymphocyte (CTL) assay

Details of the method used in this assay have been previously described [35]. Briefly, DC (5 × 10^5^) cultured from monocytes of HLA-A24+ donors were loaded with 10 μM Epstein-Barr virus (EBV)-derived peptide (TYGPVFMCL) for 2 h at 37°C in 5% CO_2_. EBV-derived peptide can bind to HLA-A2402 as reported by Lee et al. [36]. In 24 well plates, autologous CD8+T cells were co-cultured as effector cells at a ratio of 2:1 with DC in 2 ml of medium (RPMI 1640:AIM-V = 1:1) containing 10% FCS (Sigma; St. Louis, MO), 1% penicillin-streptomycin, and 0.1 mM nonessential amino acid supplemented with 100 U/ml IL-2 for 10 days; half the medium was changed every 3 days. BEC-2 cell lines (HLA-A2402) and BamB-2 cell lines (HLA-A1/A26) were generated from EBV-transformed B-lymphoblastoid cell lines which were generated from an EBV + healthy donor. The BEC-2 cells and Bamb-2 cells as target cells were loaded at a concentration of 1 × 10^6^ cells/ml with 10μM EBV peptide and incubated for 2 h at 37 °C in 5% CO_2_. Effector cells were co-cultured with target cells at effector-to-target ratios of 2:1, 10:1 and 20:1 in 96 well round-bottomed culture plates (total volume of 200 μl) in duplicate. After overnight incubation at 37 °C in 5% CO_2_, IFN-γ-concentration of the supernatant was determined by ELISA.

### ^51^Cr release assay

Details of the methods used in this assay have been previously described [37]. Briefly, effector cells were incubated with ^51^Cr labelled target cells (1 × 10^4^) at various effecor-to-target cell ratios for 6 h, followed by supernatant harvesting for measuring radioactivity using as automatic γ counter. This assay was done in triplicate.

### Statistical analysis

Statistical analysis of the results was performed by ANOVA. Differences were considered statistically significant when p value were less than 0.05.

## Results

### Phenotype and endocytic capacity

To study the direct effects of M1, M4, T-cadinol and calamenene on the maturation of sentinel DC into effector DC, human monocytes-derived DC were cultured with M1, M4, T-cadinol or calamenene. Human monocytes were cultured with GM-CSF and IL-4 for 6 days under standard conditions, followed by another 2 days in the presence of various concentrations of M1, M4, T-cadinol or calamenene. LPS, CT and TNF-α were used as a positive control. The resulting populations of DC were analyzed by flow cytometry. As shown in Table 1, the expression levels of CD1a, CD80, CD83, CD86 and HLA-DR as expressed by MFIon M1-primed DC, M4-primed DC, T-cadinol-primed DC and calamenene-primed DC were enhanced in a dose-dependent manner. The expression level of CD14 as expressed by MFIon day 8 was low or undetectable. Viability of cells at 20 μM of M1 and M4, and at 10 μM of T-cadinol and calamenene was >95%. From these results, the concentrations of M1 and M4 were used at 20 μM, and of T-cadinol and calamenene at 10 μM, respectively. The expression levels of CD1a, CD 80, CD83, CD86 and HLA-DR as expressed by MFIon LPS-primed DC, CT-primed DC, and TNF-α-primed DC were also enhanced (Table1). This pattern was identical with that of M1-primed DC, M4-primed DC, T-cadinol-primed DC or calamenene-primed DC. As control, immature DC were generated by cultivating human monocytes with GM-CSF and IL-4 for 8 days. This differentiation process is accompanied by changes in the expression of several surface markers as detailed in Table 1. Upon maturation and concomitant with an increase in Ag presenting function, DC have a reduced capacity for Ag capture via endocytic activity. To determine whether mechanisms of Ag capture could also be modulated by M1, M4, T-cadinol or calamenene, the endocytic activity was measured in immature DC, M1-primed DC, M4-primed DC, T-cadinol-primed DC or calamenene-primed DC. FITC-dextran uptake mediated by M1-primed DC, M4-primed DC, T-cadinol-primed DC, calamenene-primed DC, LPS-primed DC, CT-primed or TNF-α-primed DC was significantly lower than immature DC (data not shown). These results suggested that DC differentiated by M1, M4, T-cadinol or calamenene down-regulated their endocytic capacity.

### IL-12 p70, IL-6 and IL-10 release by activated DC

**Figure 2:**
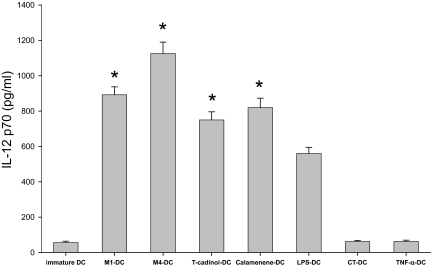

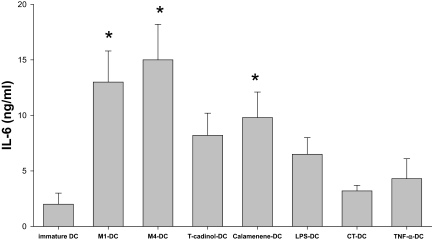

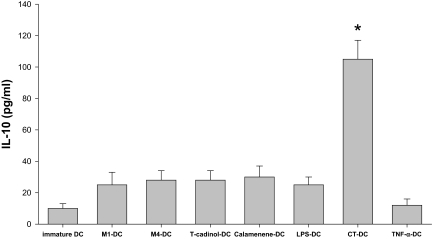
Cytokine production by CD40-L-stimulated DC DC were generated by stimulating immature DC with M1 (20 μM), M4 (20 μM), T-cadinol (10 μM), calamenene (10 μM), LPS (100 ng/ml), CT (10 μg/ml) or TNF-α (25 ng/ml). Cells (4 × 10^4^ cell/well) were stimulated with the CD40-L (3.0 μg/ml) for 24 h. After 24 h, the production of IL-12p70 (**A**), IL-6 (**B**) and IL-10 (**C**) was measured by ELISA in culture supernatants. Data are the mean ± S.E.M. of five independent experiments. *P < 0.05 compared with LPS-primed DC.

Because the level of IL-12 production by DC is a major factor driving the development of Th1 cells, we studied the influence of M1, M4, T-cadinol or calamenene on IL-12 production by DC. We measured IL-12p70 production in immature DC and in DC matured for 2 days in the presence of the above factors after stimulation by CD40-L for 24 h. The production of IL-12p70 by M1-primed DC, M4-primed DC, T-cadinol-primed DC and calamenene-primed DC was more augmented than that of LPS-primed DC (Fig. 2A). In contrast, the production of IL-12p70 by CT-primed DC or TNF-α-primed DC was low or just detectable (Fig. 2A). IL-6 is an important mediator of a wide range of biologic activities that play a critical role in the induction of proinflammatory and immune responses. Moreover, IL-10 is a pleiotropic cytokine known to have inhibitory effects on the accessory functions of DC and appears to play a central role in preventing overly pathological Th1 or Th2 responses in a variety of settings. Therefore, we also studied the production of IL-6 and IL-10 by DC differentiated under the influence of the above factors. The production of IL-6 by M1-primed DC, M4-primed DC and calamenene-primed DC was higher than that of LPS-primed DC (Fig. 2B). On the other hand, the production of IL-10 by M1-primed DC, M4-primed DC, T-cadinol-primed DC, calamenene-primed DC, LPS-primed DC and TNF-α-primed DC was low (Fig. 2C). Major enhancements of IL-10 production were caused by CT-primed DC (Fig. 2C). The production of IL-12p70, IL-6 and IL-10 was lower in immature DC (Fig. 2A, B and C).

### Immunostimulatory capacity in an allo MLR

**Figure 3:**
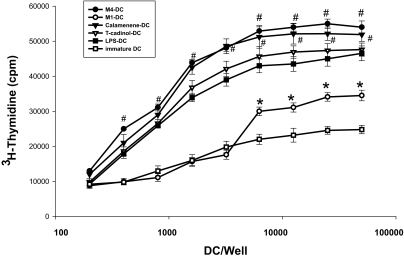
Allogeneic T cell stimulatory capacity of DC differentiated with M1, M4, T-cadinol, calamenene or LPS CD4+naïve T cells (5 × 10^4^ cell/well) were co-cultured with graded doses of M1-, M4-, T-cadinol-, calamenene- or LPS-primed DC, and on day 5, [^3^H]-methylthymidine was added 16 h before measurement of the proliferation response. Data are the mean cpm ± S.E.M. of five independent experiments. *P < 0.05 compared with immature DC; #P < 0.05 compared with LPS-primed DC.

We observed that M1-primed DC, M4-primed DC, T-cadinol-primed DC and calamenene-primed DC expressed increased levels of Ag-presenting and the expression levels of CD1a, CD80, CD83, CD86 and HLA-DR as expressed by MFI. Therefore, we next compared the capacity of M1-primed DC, M4-primed DC, T-cadinol-primed DC, calamenene-primed DC, LPS-primed DC, CT-primed DC or TNF-α-primed DC to stimulate T cells in an allo MLR. M4-primed DC showed higher stimulatory efficiency in an allo MLR than LPS-primed DC (Fig. 3), CT-primed DC or TNF-α-primed DC (data not shown). On the other hand, T cell stimulatory capacity of immature DC in an allo MLR was low (Fig. 3).

### Terpenoids-primed DC promote the differentiation of naïve T cells into Th1 cells at 1:5 DC/T cell ratio

**Figure 4:**
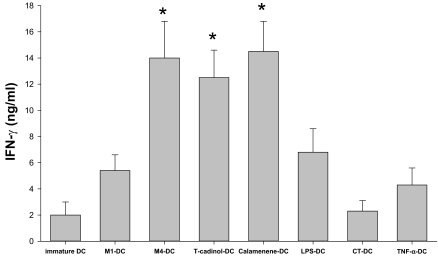

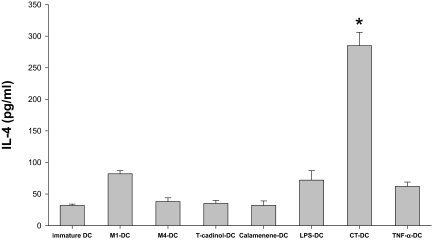
Measurements of IFN-γ and IL-4 by ELISA in supernatant of naïve T cells stimulated by M1-, M4-, T-cadinol-, calamenene-, LPS-, CT-, or TNF-α-primed DC. Allogeneic DC were co-cultured for 5 days with naïve T cells at 1:5 DC/T cell ratio. After 9 days of expansion in IL-2, T cells were counted and re-stimulated for 24 h on plates coated with anti-CD3/CD28. IFN-γ (**A**) and IL-4 (**B**) were measured by ELISA in culture supernatants. Data are the mean ± S.E.M. of five independent experiments. *P < 0.05 compared with LPS-primed DC.

**Figure 5:**
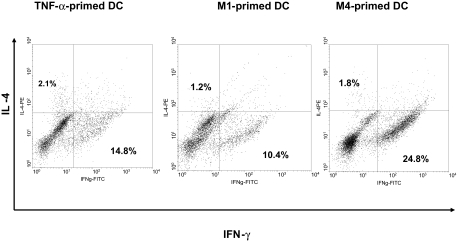
M1-, M4- and TNF-α-primed DC induced the differentiation of naïve T cells to a Th1 response at 1:5 DC/T cell ratio After 9 days of expansion in IL-2 expansion, intracellular cytokine (IFN-γ and IL-4) concentrations were measured after re-stimulation with PMA and ionomycin for 5 h. Data are one experiment representative of four independent experiments.

**Figure 6:**
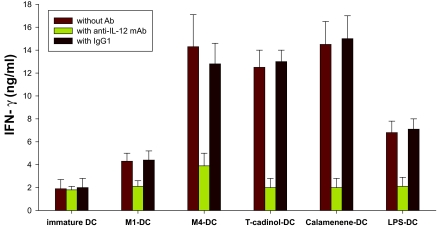

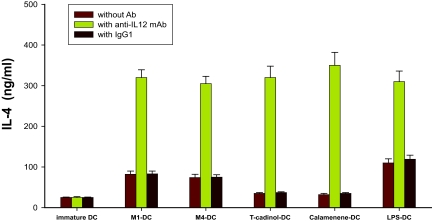
Effect of anti-IL-12 mAb on Th1 development induced by M1-, M4-, T-cadinol-, calamenene- or LPS-primed DC Allogeneic DC were co-cultured with naïve T cells at 1:5 DC/T cell ratio in the presence of control Ab or anti-IL-12 mAb (10 μg/ml). After 9 days of expansion in IL-2 expansion, T cells were counted and re-stimulated for 24 h on plates coated with anti-CD3/CD28. After 24 h, IFN-γ (**A**) and IL-4 (**B**) was measured by ELISA in culture supernatants. Data are the mean ± S.E.M. of five independent experiments.

We next evaluated the nature of primary allogeneic T cell responses stimulated by M1-primed DC, M4-primed DC, T-cadinol-primed DC and calamenene-primed DC. Allogeneic M1-primed DC, M4-primed DC, T-cadinol-primed DC or calamenene-primed DC co-cultured with naïve T cells at 1:5 DC/T cell ratio secreted sizeable amounts of IFN-γ (Fig. 4A), but little IL-4 (Fig. 4B). Similar results were obtained with LPS-primed DC and TNF-α-primed DC (Fig. 4A and B). The production of IFN-γ by naïve T cells co-cultured with M4-primed DC, T-cadinol-primed DC or calamenene-primed DC was higher than that of LPS-primed DC (Fig. 4A). Naïve T cells co-cultured with M1-primed DC, M4-primed DC, T-cadinol-primed DC, calamenene-primed DC, LPS-primed DC or TNF-α-primed DC turned into typical Th1 cells, which produced large quantities of IFN-γ and released small amounts of IL-4. In contrast, naïve T cells co-cultured with DC differentiated with CT turned into Th2 cells producing large quantities of IL-4 and releasing small amounts of IFN-γ (Fig. 4A and B). These responses were confirmed by flow cytometry (Fig. 5). On the other hand, naïve T cells co-cultured with immature DC secreted significantly less IFN-γ and IL-4 (Fig. 4A and B). To analyze the contribution of DC-derived IL-12 on the development of Th1 cells, we tested the effect of a neutralizing anti-IL12 mAb in co-culture experiments, where naïve T cells were co-cultured with M1-primed DC, M4-primed DC, T-cadinol-primed DC or calamenene-primed DC. In M1-primed DC, M4-primed DC, T-cadinol-primed DC and calamenene-primed DC, neutralization of IL-12 increased the development of IL-4 producing T cells and dramatically decreased the development of IFN-γ producing T cells (Fig. 6A and B). Similar results were obtained with LPS-primed DC (Fig. 6A and B). In contrast, the isotype control had no effect (Fig. 6A and B).

### Terpenoids-primed DC are capable of migration *in vitro*

**Figure 7:**
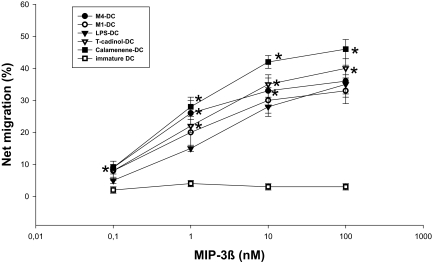

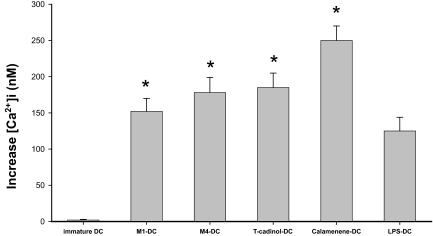

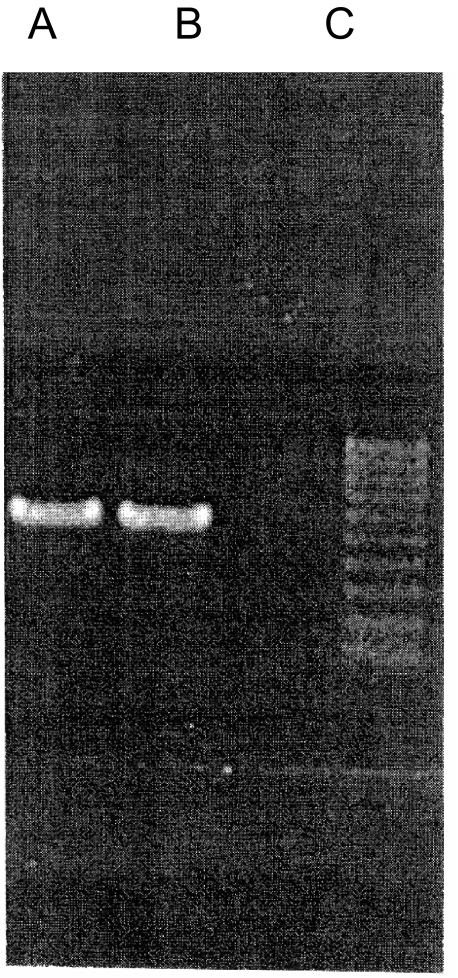
Chemotaxis and intracellular Ca^2+^ mobilization in response to MIP-3β by M1-, M4-, T-cadinol-, calamenene- or LPS-primed DC. (**A**) M1-, M4-, T-cadinol-, calamenene- or LPS-primed DC were prepared and recovered, and their migratory abilities in response to MIP-3β (0.1–100 nM) were determined *in vitro*. Data are the mean ± S.E.M. of five independent experiments. *P < 0.05 compared with LPS-primed DC. (**B**) [Ca^2+^]i mobilization of M1-, M4-, T-cadinol-, calamenene- or LPS-primed DC induced by MIP-3β (100 nM). Cells were loaded with fura-2/AM and the ratio of fluorescence at 340 nm and 380 nm was monitor. Data are the mean ± S.E.M. of five independent experiments. *P < 0.05 compared with LPS-primed DC. (**C**) cDNA prepared from T-cadinol-primed DC and calamenene-primed DC was subjected to RT-PCR-specific primer. The PCR products (573 bp) were fractionated on 2% agarose gels and visualized by ethidium bromide staining. a: T-cadinol-primed DC, b: calamenene-primed DC, c: immature DC.

Because during the maturation process, DC up-regulate the synthesis of constitutive chemokines and the CCR7 receptor, we measured the migration of M1-primed DC, M4-primed DC, T-cadinol-primed DC, calamenene-primed DC, or LPS-primed DC in response to MIP-3β *in vitro*. T-cadinol-primed DC and calamenene-primed DC had a higher migration response to MIP-3β than LPS-primed DC (Fig. 7A). Similar results were obtained with intracellular Ca^2+^ mobilization to MIP-3β (Fig. 7B). Migration response and intracellular Ca^2+^ mobilization to MIP-3β were observed with CT-primed DC and TNF-α-primed DC (data not shown). On the other hand, migration response and intracellular Ca^2+^ mobilization to MIP-3β in immature DC were low or just detectable (Fig. 7A and B). Additionally, RT-PCR confirmed the up-regulation of the CCR7 receptor in T-cadinol-primed DC and calamenene-primed DC (Fig. 7C). Similar results were obtained with M1-primed DC and M4-primed DC (data not shown).

### DC cultured with M4 augment the cytotoxicity of CD8+T cells against BEC-2 target cells

**Figure 8:**
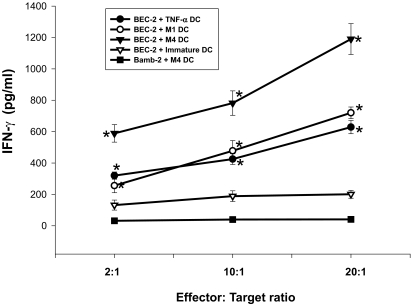

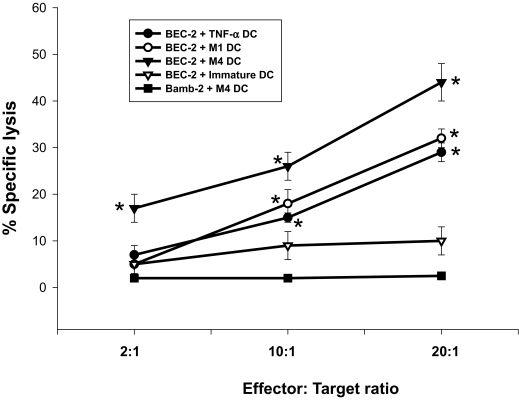
Autologus CD8+T cells with DC showed higher cytolytic activity against BEC-2 target cells at a high effector-to-target ratio than against Bamb-2 target cells. Autologous CD8+T cells were co-cultured with M1-, M4- or TNF-α-primed DC loaded with EBV-derived peptide, expanded with 100 U/ml IL-2, and on day 10, re-stimulated with BEC-2 or Bamb-2 target cells at various effector-to-target ratios. After overnight culture, the IFN-γ concentration in the supernatant was measured by ELISA (**A**), and specific lysis was measured by ^51^Cr release assay (**B**). Data are the mean ± S.E.M. of five independent experiments. *P < 0.05 compared with immature DC.

We compared the CTL responses of autologus CD8+T cells supported DC differentiated with M1, M4 or TNF-α. The production of IFN-γ by M4-primed DC was strongly augmented (Fig. 8A). The production of IFN-γ on M1-primed DC was more augmented than that of immature DC (Fig. 8A). The production of IFN-γ was dependent on the increased number of effector cells. Similar results were obtained with ^51^Cr release assay to measure specific lysis of target cells (Fig. 8B), On the other hand, the production of IFN-γ and ^51^Cr release was not observed when BamB2 (HLA-A24−) was used as a target cell (negative control) (Fig. 8A and B).

## Discussion

The present study was performed in order to investigate whether M1 and M4, end products of steroidal ginseng saponins metabolized in digestive tracts, as well as T-cadinol and calamenene can change the phenotype and function associated with DC differentiation from human monocytes *in vitro*. Here, we demonstrate that culture of immature DC with M1, M4, T-cadinol or calamenene increase cell surface expression of CD1a, CD80, CD83, CD86 and HLA-DR, while decreasing endocytic activity, resulted in cells with a phenotype characterized by efficient Ag-presentation and costimulatory capacity of mature DC. Functionally, M1-primed DC, M4-primed DC, T-cadinol-primed DC or calamenene-primed DC have enhanced primary allogeneic T cell stimulatory activity in an allo MLR.

The differentiation of monocytes into DC has a critical impact on the immune response. DC interaction with naïve T cells plays a key role in primary immune responses [1, 3] and the interaction of T cells with DC is crucial for directing T cell differentiation towards the Th1, Th2 or Th17 type [18, 38]. The balance of Th1 and Th2 cells is characterized by different cytokine production and homing capacity [10–12], and strongly depends on the model system used [6, 7]. M1-primed DC, M4-primed DC, T-cadinol-primed DC or calamenene-primed DC polarized into Th1 via high IL-12p70 secretion upon CD40-L (T cells engagement) stimulation and demonstrated that the production of IFN-γ by naïve T cells co-cultured with M1-primed DC, M4-primed DC, T-cadinol-primed DC or calamenene-primed DC was affected by the presence of a neutralizing anti-IL-12 mAb. The reduced induction of IFN-γ after incubation with anti-IL-12 mAb indicates that IFN-γ induction is largely dependent on endogenous IL-12. Cytokines are most important in the environment response, and IL-12 is essential for inducing Th1 polarization [39, 40]. IL-12 has anti-tumor effects of its own for which it is currently under clinical evaluation [39]. Therefore, the finding that M1, M4, T-cadinol or calamenene can increase IL-12p70 secretion by DC is of major interest, although the rational for ginseng saponins use in the treatment of cancer is still unclear. Although the down-regulation of NF-κB and AP-1 transcription factors has often been hypothesized to explain the anti-cancer effects of ginseng saponins, there is some evidence that these mechanisms do not clearly account for the anti-cancer effect of ginseng saponins [41]. Wakabayashi et al. [42] have demonstrated that the induction of an *in vivo* anti-metastatic effect by oral administration of ginsenosides may be primarily meditated by their metabolic component M1 and M4. Moreover, Scaglione et al. [43] have investigated immunomodulatory effects of ginseng extract G115 and they have indicated an earlier induction immune response mediated by their G115 preparation than by the aqueous extract. The Th1 cells that produce IFN-γ have been shown to exert a powerful anti-tumor effect, whereas a weak Th1 or a Th2 profile may have an opposite effect, that is, down-regulation of innate and acquired anti-tumor immunity [44]. The corollary of these observations is that a Th1 profile may be protective against tumor growth and dissemination. Moreover, a recent study describes mycoplasma as another pathogen involved in DC activation, resulting in release of IL-12, TNF-α and IL-6 [44]. M1-primed DC, M4-primed DC, T-cadinol-primed DC or calamenene-primed DC provide stronger costimulatory signals and/or the proinflammatory cytokines needed for T cell activation and Th1 development. Therefore, it suggests that the effects of M1 or M4 on the production of IL-12p70 by DC and strengthening of the Th1 response by naïve T cells might, at least in part, contribute to a potential anti-tumor effect of M1 or M4. Ginseng may be the most commonly used diet supplement in the world. It is unlikely that healthy individual develop harmful self-destructive Th1 response by ginseng, because there are few the content of ginseng in diet supplement. It seems that TNF-α-primed DC drive the differentiation of naïve T cells towards Th1 via an unknown factor, because TNF-α-primed DC did not increase the IL-12 production upon CD40 ligation.

In contrast, CT-primed DC induced naïve T cells showed a shift towards Th2 effector cell. CT is a powerful mucosal adjuvant that amplifies B cell and T cell responses to mucosally co-administered antigens, stimulating predominant Th2-type responses [45, 46]. Gagliardi et al. [48] have demonstrated that CT-treated DC induce Th2 polarization of naïve T cells *in vitro* is very likely due to the lack of IL-12 production by CT-treated DC and may be independent of IL-4. We showed that the production of IL-12 by CT-primed DC that were stimulated CD40-L was low or detectable. Therefore, it suggests that the involvement of these cells in the innate responses to Th1 response-inducing pathogens during the period preceding the initiation of adaptive immunity can create an environment rich in IL-12, which promotes the production of IFN-γ and the outgrowth of Th1 cells. It has been demonstrated that immune balance controlled by cytokines such as IL-10 and IL-12 plays an important role in immune regulation, including anti-tumor immunity [16]. Our results showed that M1-primed DC, M4-primed DC, T-cadinol-primed DC and calamenene-primed DC have a decreased ability to produce IL-10, and that these DC have the potency to induce Th1 cells. In contrast, the production of IL-10 by CT-primed DC was high and these DC polarized into Th2 cells. Therefore, it seems likely that the development of the Th1 response is controlled, at least partly, by IL-10 and IL-12 and may be a crucial polarizing factor for Th1 response development.

One result of DC maturation and activation demonstrate increased motility and a potential to propensity to migrate towards lymphoid organs. During the maturation process, DC down-regulate the expression of inflammatory chemokines and their receptors and up-regulate the synthesis of constitutive chemokines and the chemokine receptor CCR7. We found that M1-primed DC, M4-primed DC, T-cadinol-primed DC and calamenene-primed DC had high migration, high calcium mobilization in response to MIP-3β and up-regulated the expression of CCR7, suggesting that M1-primed DC, M4-primed DC, T-cadinol-primed DC and calamenene-primed DC are likely to be mature DC that have the potential to migrate *in vivo*. In most clinical trials using DC-based immunotherapy, immature monocytes-derived DC pulsed with tumor antigen peptides were used. However, recent observation suggests that mature DC could be a better anti-tumor adjuvant [49]. Although the role of chemokine receptor for clinical application are not known yet, M1, M4, T-cadinol and calamenene appear to be a good factor to induce DC maturation, or even better in some respect, for the use in clinical DC therapy to induce strong Th1-type immune responses. The concentration of terpenoids in this in vitro used 10 μM, but this concentration achievable in any in vivo are still not clear. The experiment of in vivo on terpenoids-primed DC requires further investigation.

Antitumor immunity has classically been measured by the quantity of tumor-antigen-specific CD8+ T cells [50]. Immunotherapy is based on this mechanism. In this context, it is important to know whether M1-primed DC or M4-primed DC enhanced specific CTL responses. We evaluated the CTL response to examine the possible clinical application of our culture system, since DC differentiated with M1 or M4 induced a significant differentiation of naïve T cells towards a helper T cells type I response. As expected from their Th1-polarizing effect, the DC differentiated with M4 induced a stronger CTL response than immature DC and TNF-α-primed DC. Therefore, we are expecting the possibility of a clinical application. The effects of T-cadinol and calamenene in the CTL response are not known yet. However, we expect to obtain similar results with M1 and M4, because T-cadinol-primed DC and calamenene-primed DC enhanced the differentiation of naïve T cells towards the Th1 type. Recent studies have demonstrated that the ginseng saponins orally administrated are promptly metabolized in the digestive tract and absorbed into the circulation [23, 24]. The major metabolites of the proto-panaxidiol saponin are M1, M2, M3 and M5, and those of the protopanaxatriol are M4 and M11 (Fig. 1). Finally, the protopanaxadiol and the protopanaxatriol saponins and their intermediate metabolites (M2, M3, M5 and M11) are converted into the end products M1 protopanaxadiols and M4 protopanaxatriol, respectively. There is the possibility that the metabolites are the actual active substances of the ginseng saponins, particularly M1 and M4, *in vivo*.

## Conclusion

This is first study on the effect of terpenoids, e.g. ginseng saponins on human monocytes-derived DC giving new insights into the action of terpenoids. M1, M4, T-cadinol and calamenene potentially regulate differentiation of DC from human monocytes in combination with GM-CSF and IL-4 *in vitro*. Moreover, DC differentiated with M1, M4, T-cadinol or calamenene enhance the differentiation of naïve T cells towards the Th1 type depending on IL-12 secretion. M-4-primed DC produces a stronger CTL response. Although the molecular events leading to the effects of M1, M4, T-cadinol and calamenene on DC function remain to be resolved, DC appear to be potential targets of M1, M4, T-cadinol and calamenene. Further understanding of the mechanisms by which M1, M4, T-cadinol and calamenene modulate DC function may lead to the development of effective immunotherapy for cancer.

## Figures and Tables

**Table 1:** Comparison phenotype of DC cultured with M1, M2, T-cadinol and Calamenene on day 8.

	CD1a	CD80	CD83	CD86	HLA-DR
M1					
1 μM	62 ± 19	109 ± 13	48 ± 13	398 ± 12	989 ± 86
10 μM	198 ± 25	148 ± 7	62 ± 18	458 ± 35	1081 ± 63
20 μM	183 ± 12	203 ± 14	82 ± 12	560 ± 34	1190 ± 132
M4					
1 μM	81 ± 32	102 ± 6	48 ± 4	348 ± 38	1056 ± 85
10 μM	122 ± 18	148 ± 6	69 ± 9	465 ± 15	1189 ± 142
20 μM	212 ± 40	208 ± 6	106 ± 32	592 ± 48	1334 ± 105
T-cadinal					
0.1 μM	65 ± 18	110 ± 28	32 ± 12	331 ± 38	834 ± 76
1.0 μM	157 ± 21	159 ± 29	56 ± 18	452 ± 36	921 ± 98
10 μM	183 ± 22	208 ± 32	81 ± 19	586 ± 41	1058 ± 121
Calamenene					
0.1 μM	78 ± 19	105 ± 28	35 ± 13	335 ± 39	956 ± 108
1.0 μM	167 ± 29	158 ± 31	63 ± 16	443 ± 41	1168 ± 231
10 μM	210 ± 31	221 ± 33	109 ± 21	601 ± 38	1267 ± 298
LPS(100 ng/ml)	201 ± 20	232 ± 36	106 ± 20	622 ± 41	1308 ± 299
CT(10 μg/ml)	209 ± 18	228 ± 32	68 ± 8	680 ± 82	1412 ± 389
TNF-α (25 ng/ml)	196 ± 48	209 ± 9	82 ± 18	620 ± 43	1395 ± 125
immature DC	80 ± 10	48 ± 4	9 ± 2	334 ± 28	665 ± 98
Monocyte	5 ± 1	5 ± 2	6 ± 2	11 ± 2	56 ± 3

Data are the mean ± S.E.M. of five independent experiment.

## Abbreviations

DC: dendritic cells
CTL: cytotoxic T-lymphocyte assay
MLR: allogeneic mixed lymphocyte reaction
LPS: lypopolysaccharide
TNF-α: tumor necrosis factor-alpha
GM-CSF: granulocyte-macrophage colony-stimulation factor
IFN-γ: interferon-gamma
MFI: mean fluorescence intensity
PE: phycoerythrin
FITC: fluorescent isothiocyanate
EBV: Epstein-Barr virus
Ag: antigen
